# Improvement of APACHE II score system for disease severity based on XGBoost algorithm

**DOI:** 10.1186/s12911-021-01591-x

**Published:** 2021-08-06

**Authors:** Yan Luo, Zhiyu Wang, Cong Wang

**Affiliations:** 1 Present Address: School of Computer Science (National Pilot Software Engineering School) , Beijing University of Posts and Telecommunications, Beijing, 100876 China; 2 Key Laboratory of Trustworthy Distributed Computing and Service (BUPT), Ministry of Education, Beijing, 100876 China

**Keywords:** APACHE II score system, Machine learning, Predictive modeling, MIMIC III database, Intensive care units treatment

## Abstract

**Background:**

Prognostication is an essential tool for risk adjustment and decision making in the intensive care units (ICUs). In order to improve patient outcomes, we have been trying to develop a more effective model than Acute Physiology and Chronic Health Evaluation (APACHE) II to measure the severity of the patients in ICUs. The aim of the present study was to provide a mortality prediction model for ICUs patients, and to assess its performance relative to prediction based on the APACHE II scoring system.

**Methods:**

We used the Medical Information Mart for Intensive Care version III (MIMIC-III) database to build our model. After comparing the APACHE II with 6 typical machine learning (ML) methods, the best performing model was screened for external validation on anther independent dataset. Performance measures were calculated using cross-validation to avoid making biased assessments. The primary outcome was hospital mortality. Finally, we used TreeSHAP algorithm to explain the variable relationships in the extreme gradient boosting algorithm (XGBoost) model.

**Results:**

We picked out 14 variables with 24,777 cases to form our basic data set. When the variables were the same as those contained in the APACHE II, the accuracy of XGBoost (accuracy: 0.858) was higher than that of APACHE II (accuracy: 0.742) and other algorithms. In addition, it exhibited better calibration properties than other methods, the result in the area under the ROC curve (AUC: 0.76). we then expand the variable set by adding five new variables to improve the performance of our model. The accuracy, precision, recall, F1, and AUC of the XGBoost model increased, and were still higher than other models (0.866, 0.853, 0.870, 0.845, and 0.81, respectively). On the external validation dataset, the AUC was 0.79 and calibration properties were good.

**Conclusions:**

As compared to conventional severity scores APACHE II, our XGBoost proposal offers improved performance for predicting hospital mortality in ICUs patients. Furthermore, the TreeSHAP can help to enhance the understanding of our model by providing detailed insights into the impact of different features on the disease risk. In sum, our model could help clinicians determine prognosis and improve patient outcomes.

## Background

According to data from World Population Prospects: the 2019 Revision, there will be more than twice as many persons above 65 as children under five by 2050 [1]. Those increasing numbers of elderly patients and the emphasis on the long-term quality of life in patients with critically ill have led to a growing demand for intensive care units (ICUs). The prognosis of patients admitted to the ICUs is quite different and the mortality rate can range from 7% [2] to 52.3% [3]. It is because critical care is fast-paced, complex, and commonly requires urgent high-risk decision-making, and the outcome of ICUs treatment is highly related to numerous factors, such as the site, cause of admission, age, prior comorbidities, acute physiological changes at admission and during the first several hours of treatment, etc. [4]. Therefore, one of the many significant challenges faced by physicians is the need to deal with a tremendous amount of real-time information and make the best decision to deal with these patients. It relies heavily on physicians' workability. Moreover, the severity and instability of critically ill patients’ illness and their frequent need for high-risk interventions and medications lead to the higher rates of adverse events and medical error rates than elsewhere [5]. Rothschild et al. [6] studied 391 patients with 420 unit admissions during 1490 patient-days and found that the rates per 1000 patient-days for adverse events and serious errors which had life-threatening were 13% and 11%, respectively.

To solve these problems, many researchers have been committed to developing predictive scoring systems of measures disease severity that is used to predict outcomes, typically mortality, of patients in the ICUs [7]. There are many predictive scoring systems that can achieve an objective and quantitative description of the degree of organ dysfunction and evaluation of morbidity in ICUs patients such as Simplified Acute Physiology Score (SAPS), Sepsis-related Organ Failure Assessment Score (SOFA), Multiple Organ Dysfunction Score (MODS), Logistic Organ Dysfunction Score (LODS) and Acute Physiology and Chronic Health Evaluation (APACHE) II, III and IV. But until Knaus et al. [8] published the second version of the APACHE II in 1985, the prognostic scores for critically ill patients were established, and quickly became the most widely used prognostic index in ICUs and clinical trials worldwide.

Although APACHE III and IV have been developed, they are too complex and time-consuming for routine use in the ICUs. Therefore, APACHE II is still the most widely used severity-of-disease scoring system in ICUs around the world [9–13] and become the most cited study in the intensive medicine literature to date [14, 15]. So the purpose of this article is to make improvements based on Apache II. APACHE II is a composite score consists of Age, the Chronic Health Index (CHI) and the Acute Physiology Score (APS), and the latter derived from 12 physiologic parameters that include vital signs, arterial blood gas measurements, laboratory results from blood specimens, the Glasgow Coma Scale (GCS), and so forth. APACHE II was used to measure the severe disease by calculating the most deranged reading during each patient’s initial 24 h in ICUs. The increasing score (range 0–71) was closely correlated with the subsequent risk of hospital death for intensive care admissions [8]. Inexperience to date, few patients have exceeded 55 [16].

However, APACHE II has its own issues, such as too sophisticated scoring statistical approaches for routine use in the ICUs. It’s hard for physicians to use it with great ease to ensure that decision making is accurate and compatible with current therapeutic capabilities, and perform badly in repeated application [9]. Furthermore, the accuracy of a prognostic model generally deteriorates over time due to changes in ICU admission and discharge criteria, the increasing medical resource, and variations in the availability and effectiveness of different treatments for particular conditions [17]. As described by Soares et al. [18], because almost any ICUs today hospital mortality much lower than that expected in 1985, APACHE II should not be used as an evaluation tool in the ICUs.

Thus, researchers have been trying to develop a more accurate and timely method than APACHE II to measure the severity of the patients in intensive medicine [19–24]. They found machine learning (ML) techniques have a wide range of applications in disease prediction, and play a very important role in obtaining rapid and precise information about the nature of a patient’s health problem and alleviate the complications related to scoring systems such as APACHE (Table 1).

**Table 1:** Significant scholarly works that ML techniques to compare with the performance of APACHE II

Study	Data source	Condition	Number of patients	Machine learning algorithms	Accuracy	AUC
Samaneh Layeghian Javan et al.[19]	MIMIC III	Cardiac arrest	4611	Stacking algorithm	0.76	0.82
Min Woo Kang et al.[20]	Seoul National University Hospital	Continuous renal replacement therapy	1571	Random forest	/	0.78
Meng Hsuen Hsieh et al. [21]	Chi-Mei Medical Center	Patients with unplanned extubation in intensive care units	341	Random forest	0.88	0.91
Zhongheng Zhang et al.[22]	SAILS study and OMEGA study	Acute respiratory distress syndrome	1071	Neural network	/	0.821
Dan Assaf et al.[21]	Sheba Medical Center	Coronavirus disease (COVID-19)	162	Random forest	0.92	0.93
Grupo de Trabajo Gripe A Grave et al.[23]	GETGAG/SEMICYUC database	Severe influenza	3959	Random forest	0.83	0.82
Kuo-Ching Yuan et al.[24]	Taipei Medical University Hospital	Sepsis	434	XGBoost	0. 82	0.89
Scherpf M et al.[39]	MIMIC III	Sepsis	1050	Recurrent neural network	/	0.81
Zhang Z et al.[40]	MIMIC III	Acute kidney injury	6682	XGBoost	/	0.86
Kong G et al.[41]	MIMIC III	Sepsis	16,688	Gradient boosting machine		0.85
Our work	MIMIC III	ICU patients	24,777	XGBoost	0.87	0.81

According to Table 1, many ML algorithms have been used to evaluate the performance of APACHE II, but most of the research data is small. In addition, most researches have only studied a single disease, and there was no evidence that one of studies could individually outperform all others regardless of the data set. In this paper, we focused on ML algorithm selection and tuning to improve the performance of the APACHE II scoring system, by using two large data sets for modeling and validation, respectively. Those contributions were highlighted as follows:

Firstly, we compared the APACHE II scoring system with several ML models on large ICUs databases. Then, optimized these algorithms and selected the best one-eXtreme Gradient Boosting (XGBoost) to validate on another large non-overlapping ICUs database. Both databases containing ICUs data routinely collected from adult patients in the United States. The performance of XGBoost algorithm was more effective and stable than other approaches. Furthermore, we filtered and added 5 features from ICUs disease scoring scales such as Apache IV, SOFA, and SAPS to develop our model, which might provide a way to improve the APACHE II scoring system with a more efficient performance.

In recent years, prediction models such as support vector machines (SVM), logistic regression models (LR), Naïve Bayes (NB), artificial neural networks (ANN), random forest models (RF) and other machine learning models have been developed in many areas of health care research [25–29]. In this study, we aim to construct several machine learning models to predict the mortality of ICUs patients and compare their predicting performance, and finally get a prediction model that is better than the Apache II traditional scoring scale.

## Methods

### Database description and Source code

Our models were built and validated on two large non-overlapping ICUs databases, the Medical Information Mart for Intensive Care version III (MIMIC-III) version 1.4 [30] which released in September 2016 was used for model development, and the eICU Research Institute Database (eRI) [31] was used for model validation. The MIMIC-III dataset comprises of almost 60 000 inpatients treated in the ICUs of the Beth Israel Deaconess Medical Center (BIDMC) in Boston from June 2001 to October 2012. And the eICU database was a multi-center ICUs database included over 200 000 admissions monitored by eICU Programs across the United States[31]. Both data sets are publicly available after registration and contain high granularity data associated with distinct hospital admissions for ICUs adult patients in the United States, such as the real electronic medical record data of various types of ICUs (surgical intensive care unit, coronary care unit, medical intensive care unit, etc.).

With the help of the Shared resources on GitHub(https://github.com/MIT-LCP/mimic-code/tree/master/concepts/severityscores), we extracted each patient’s recorded value of the first 24 h after admission to ICUs. All source code is available in our repository under a permitted open source license to reproduce the analyses in this work(https://github.com/yannie228/MIMIC-III).

The aim of the present study is to provide ICUs patients with a machine learning algorithm model that performs better than APACHE II scoring system in evaluating and predicting disease severity. Considering that the median hospital length of stay for ICUs patients in our basic data set was 43 (0–87) days, we selected the 90-day in-hospital mortality as an outcome variable to maximize sample inclusion. Finally, after exclusion of ineligible cases (values less than 0 or outside the measuring range of the inspection device), we included 24,777 admissions (including 15.97% deaths) from MIMIC-III database and 7328 admissions (including 22.30% deaths) from eRI database, respectively. Patient demographics and clinical characteristics are shown in Table 2. In both datasets, we extracted a set of 14 variables, including demographics, vital signs, laboratory values, etc.

**Table 2:** Description of the Acute Physiology variables

variables	MIMIC III		eRI	
	Survivor n = 20,821	Non-survivor n = 3956	Survivor n = 5694	Non-survivor n = 1634
*Age*	66.00(53.51)		62.00(18.83)	
*APS*				
T(℃)	36.94(1.31)		36.40(5.03)	
MAP(mmHg)	60.00(39.70)		61.00(47.14)	
HR(bpm)	99.00(31.52)		114(31.65)	
RR(/min)	26.00(10.79)		31.00(15.83)	
A-aDO_2_(mmHg)	101.00(43.77)		100.00(86.76)	
PH	7.35(0.13)		7.36(0.11)	
Na(mmol/L)	140.00(5.85)		138.00(6.49)	
K(mmol/L)	4.40(1.16)		4.30(1.20)	
BUN(mg/dl)	26.00(26.08)		24.00(23.88)	
Ht(%)	29.20(7.43)		31.90(7.43)	
WBC(*10^9^/L)	11.50(14.59)		12.4(10.34)	
GCS(15-GCS)	1.00(2.51)		5.00(4.73)	

Figures represent median (standard deviation) for numerical variables, all values are calculated from all non-missing data, after removal of ineligible cases, and before imputation of missing data

Abbreviations: APS Acute Physiology Score, T Temperature, MAP mean arterial pressure, HR Heart Rate, RR Respiratory Rate, A-aDO_2_ alveolar-arterial differences for O_2_, PH Potential of Hydrogen, Na Sodium, K Potassium, BUN Blood Urea Nitrogen, Ht Hematocrit, WBC White Blood Cell, GCS the Glasgow Coma Scale

### Feature variable screening

The first issue in the model building via ML is feature selection. We extracted the features from MIMIC III database according to the APACHE II scoring system, and picked out the worst values within the initial 24 h after each patient been hospitalized to the ICUs, then graded them. All patients were adults (above 16 years old) and diagnosed with chronic diseases, including chronic obstructive pulmonary disease (COPD), Acquired Immune Deficiency Syndrome(AIDS), lymphoma, leukemia, etc. Finally, we have picked out 14 variables to form our basic data set, which is composed of 12 Acute Physiology variables (Table 2), age, and chronic health status.

### Data preprocessing

Data preprocessing were driven by certain qualities of our data set, so we normalized and standardized the data based on APACHE II scores. As more than half of the variable A-aDO_2_ were missing, after removing the case with missing values in the variables except for A-aDO_2_, we used the RF classifier to fill in the missing values of A-aDO_2_. At last, we included 21 940 admissions and 6 893 admissions from MIMIC-III and eRI, respectively. Normalization is a "scaling down" transformation of the features [32]. Because the value range of the original data in the data set that used in this article has been determined, in order to preserve the relationship among the original data values, we chose the following Min–max normalization method to normalize our data.

1 $${z_{ij}} = \frac{{{x_{ij}} - \min ({x_{ \cdot j}})}}{{\max ({x_{ \cdot j}}) - \min ({x_{ \cdot j}})}}$$

so that the normalized data in terms of z_ij_ has the element value between 0 and 1. However, it is noticeable that data distribution is unbalanced in the overall data set: the ratio between patient death and survival is 1:5.263. To ensure that the output of the prediction model does not over-fit the data, we weighted the data according to their outcome ratios when training the models.

### Modeling approach selection

The primary outcome measure in our paper was hospital mortality. Group mortality prediction of acutely ill patients for the APACHE II score was calculated as defined by Knaus et al. [8], for each individual to compute the risk(R) of hospital death with the following equation; then sum the individual risks and divide by the total number of patients.

2 $$\begin{aligned} {\text{In}}\left( {{\text{R}}/1 - {\text{R}}} \right) & = - 3.517 + \left( {{\text{APACHE II score}} \times 0.146} \right) \\ & \quad + \left( {0.603,{\text{ only if postemergency surgery}}} \right) \\ & \quad + \left( {\text{diagnostic category weight}} \right) \\ \end{aligned}$$

However, using the estimated death rate of the individual obtained by formula 2, a classification matrix containing True Positive (TP), False Positive (FP), True Negative (TN) and False Negative (FN) was obtained with a decision criterion of 0.5, and then the accuracy, precision, recall rate, F1 and ROC values of Apache II were calculated according to the matrix. The decision criterion of 0.5 means that every patient with a risk greater than 0.5 is predicted to die [8].

**Fig. 1:**
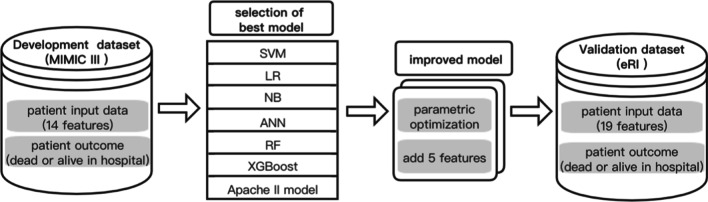
Data flow of the modeling process. The MIMIC-III data set was used for model training and testing. These 6 ML methods were compared with the traditional Apache II method to select the best model for model improvement, then external validation was performed on an independent data set from the eRI database

Meanwhile, we run a preliminary test for several typical ML methods which widely used in the binary calculation, such as SVM, LR, NB, ANN, RF and the XGBoost on our ICUs data set and evaluated their performances. Then selected the best one to build the model. The proposed modeling process was shown in Fig. 1. All ML algorithms of this research were done with the help of the scikit-learn. Scikit-learn is probably the most useful library for machine learning in Python that contains a lot of efficient tools for machine learning and statistical modeling, and data preprocessing package. The analysis was carried out on the Jupyter Notebooks platform, using the Python programming language.

**Fig. 2:**
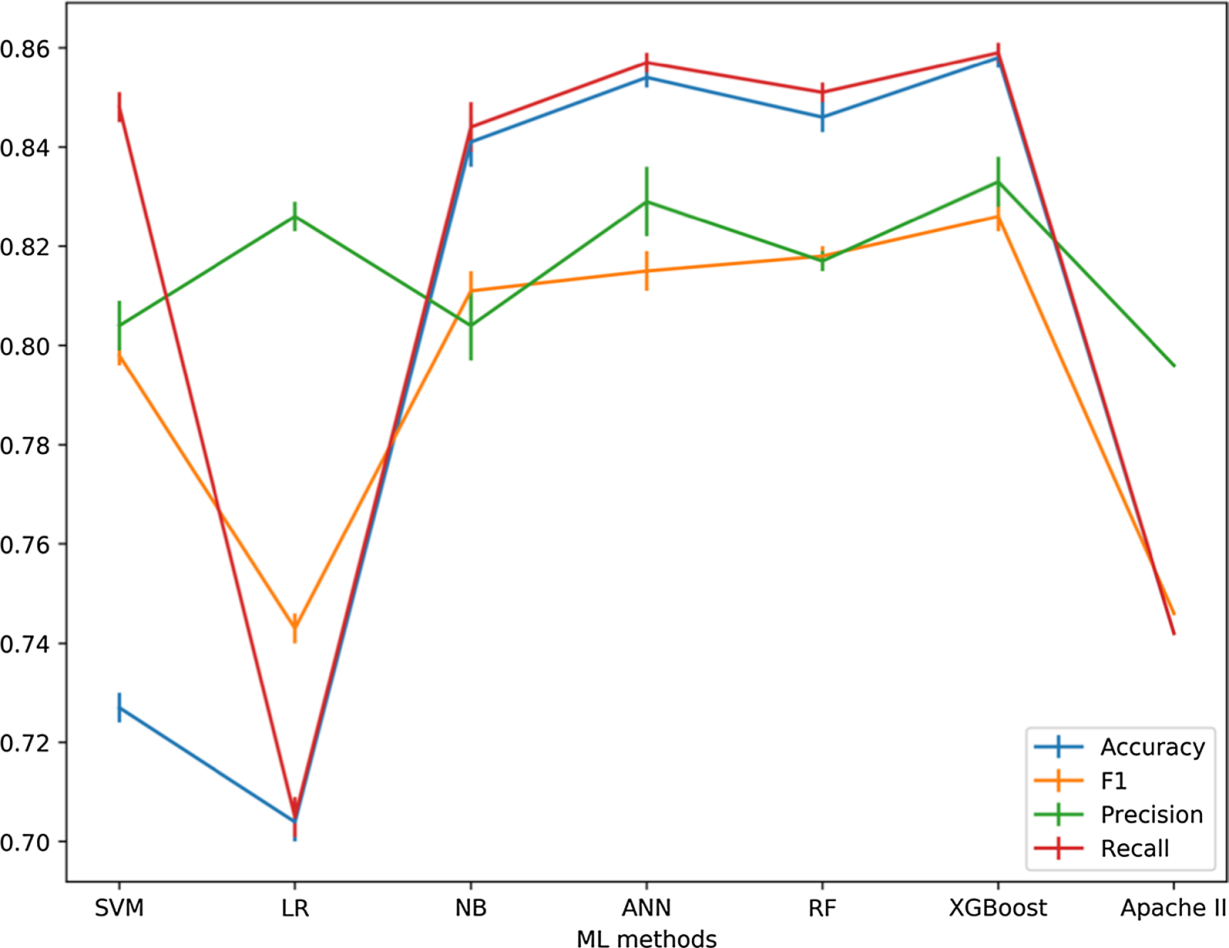
Mean ± Standard Error(SE) of Cross Validation for SVM, LR, NB, ANN, RF, XGBoost and Apache II models. The data set used for model building contains 14 feature variables

In this study, since we had labeled records, the supervised learning approach was used. And Grid Search is an effective method for adjusting the parameters in supervised learning and improve the generalization performance of a model [33]. To achieve optimal performance, we chose the GridSearchCV to adjust the parameters of the above six machine learning models (Table 3). With Grid Search, we tried all possible combinations of the parameters of interest and found the best ones. GridSearchCV is a useful tool that traverse all the different parameters that is fed into the parameter grid and produces the best combination of parameters, based on a scoring metric (accuracy, f1, etc.). According to Table 4 and Fig. 2, with Grid Search, we tried all possible combinations of parameters of interest and found XGBoost was appealing because its average performance was better than the other classifiers in our test. In this article, we chose XGBoost as an example to demonstrate the tuning process.

**Table 3:** Optimal parameter values for 6 ML models

Abbreviation	Hyperparameter	Value
SVM	C	2
	Gamma	1
LR	C	0.1
NB	Alpha	1
ANN	Hidden_layer_sizes	30
RF	n_estimators	400
XGBoost	Lerning_rate	0.1
	n_estimator	90
	Gamma	0.05
	reg_lambda	0.1
	Subsample	0.6
	Min_child_weight	1
	max_depth	5

**Table 4:** Performance of ML methods

Abbreviation	Accuracy [95% Cl]	Precision [95% Cl]	Recall [95% Cl]	F1 [95% Cl]
SVM	0.736 ± 0.003 [0.732, 0.740]	0.819 ± 0.005 [0.813, 0.825]	0.736 ± 0.003 [0.732, 0.740]	0.765 ± 0.002 [0.763, 0.767]
LR	0.704 ± 0.004 [0.698, 0.710]	0.822 ± 0.003 [0.817, 0.826]	0.704 ± 0.004 [0.698, 0.710]	0.742 ± 0.003 [0.737, 0.746]
NB	0.841 ± 0.005 [0.834, 0.847]	0.802 ± 0.007 [0.792, 0.812]	0.841 ± 0.005 [0.834, 0.847]	0.809 ± 0.004 [0.803, 0.814]
ANN	0.854 ± 0.002 [0.851, 0.858]	0.827 ± 0.007 [0.817, 0.837]	0.854 ± 0.002 [0.851, 0.858]	0.810 ± 0.004 [0.805, 0.815]
RF	0.841 ± 0.003 [0.837, 0.845]	0.840 ± 0.002 [0.837, 0.843]	0.840 ± 0.001 [0.839, 0.841]	0.841 ± 0.002 [0.838, 0.843]
XGBoost	0.858 ± 0.002 [0.855, 0.862]	0.834 ± 0.005 [0.827, 0.841]	0.858 ± 0.002 [0.855, 0.862]	0.824 ± 0.003 [0.820, 0.829]
Apache II	0.742 ± 0.000	0.796 ± 0.000	0.742 ± 0.000	0.764 ± 0.000

### XGBoost model optimization

XGBoost is a sequential technique which works on the principle of ensemble [34]. It combines a set of weak learners to improve prediction accuracy and always shows better performance on various ML benchmark data sets [35]. As mentioned above, we used the Grid Search in this article, with XGBoost as the example shown below.

Step 1: Since our study was a binary categorization model, the objective chosen “binary: logistic”. The “n_estimators” which determine the epoch of the model was set to 100 and early stopping rounds to 10 to check for overfitting.

Step 2: Search for the optimal learning_rate and gamma simultaneously because they directly affect the performance of the model. The grid values searched for the learning_rate were 0.01, 0.02, 0.1, 0.2, and 0.3, while those for the gamma were 0.0, 0.1, 0.2, 0.3 and 0.4.

Step 3: With the optimal values of the learning_rate and gamma, make a Grid Search over the max_depth and min_child_weight in selected ranges of 1 to 10. All the possible combinations of these two parameter values were run for the model tuning and the one with the best performance was retained as the optimal values.

Step 4: Make a Grid Search over the regularization parameter reg_alpha, colsample_bytree, and subsample simultaneously in their respective optimal ranges.

### Feature extraction

**Fig. 3:**
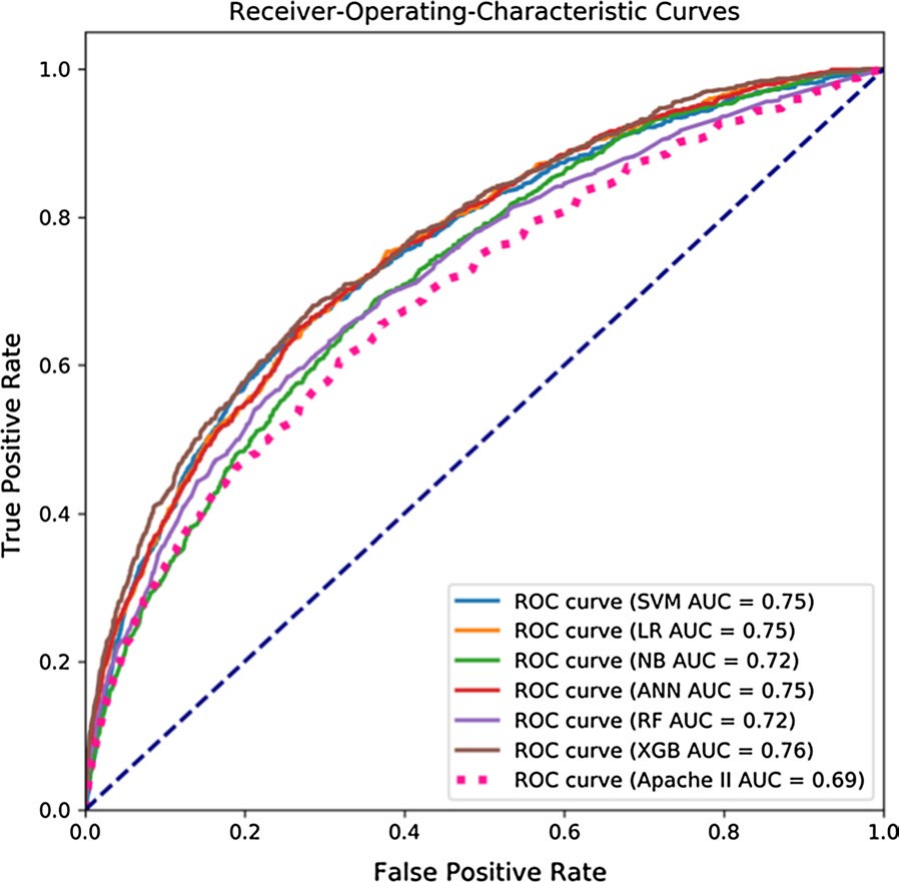
Receiver-Operating Characteristics curves of SVM, LR, NB, ANN, RF, XGB, and Apache II models. These results were obtained using fivefold cross-validation

After getting the best parameters of the six ML models, through Fig. 3 we found that when only using the same feature vectors as Apache II, the area under the curve (AUC) values of these models are all lower than 0.80. Therefore, we tried to reconstruct a new dataset by picking out some new ICUs characteristic variables from the disease severity prediction scoring systems such as Apache IV, SOFA, and SAPS to improve model performance. In the end, we selected five new variables(albumin, bilirubin, serum creatinine, urine and blood glucose) and obtained a new basic data set containing 19 variables.

**Fig. 4:**
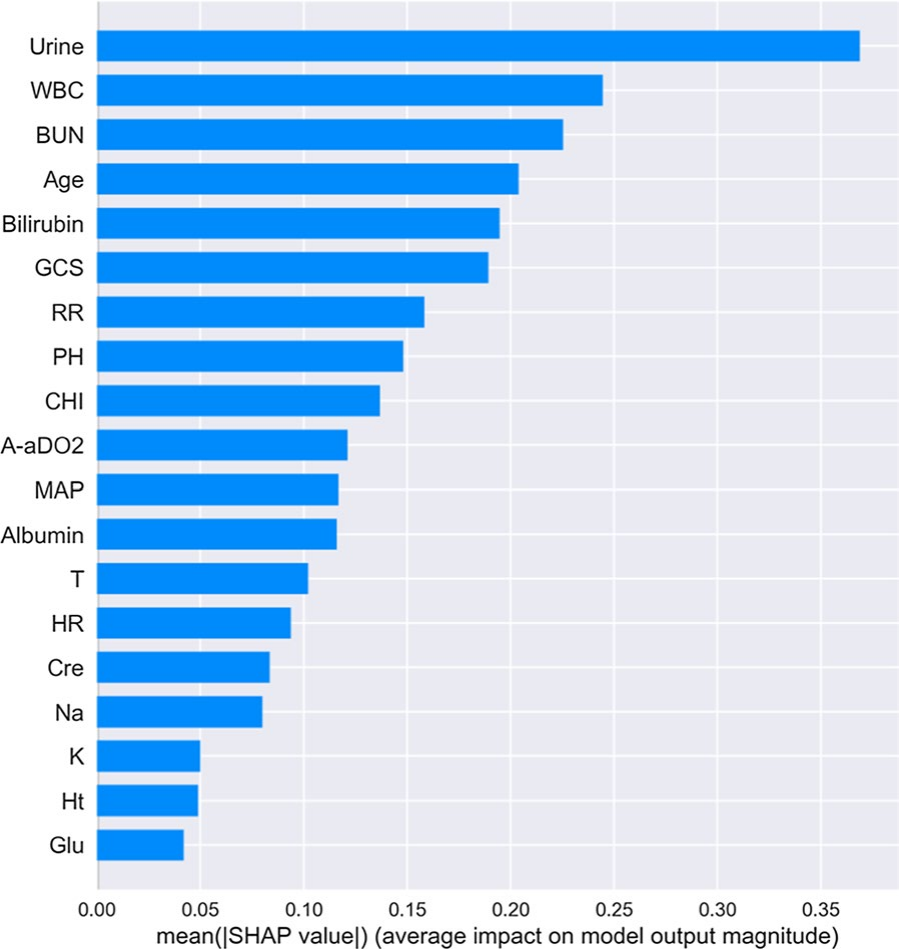
Rank of importance of each variable in the XGBoost model for intensive care unit mortality prediction

Furthermore, the XGBoost library provides a built-in function(plot_importance()) to plot features ordered by their importance. Feature importance can intuitively reflect the importance of features, and select features that have a greater impact on the final model. However, it is impossible to judge the relationship between the characteristics and the final prediction result. So we used Shapley Additive Explanations (SHAP) to gain some insight into which features contribute enough information to the model's calculations (Fig. 4). SHAP is a method to explain model predictions based on Shapley Values, with it we can explain how much each feature contributes to the value of a single prediction[36]. For testing the influence of new variables on the XGBoost model, we then sorted the five newly added variables according to Fig. 4, and included them into the dataset in order of their contribution degree for independent testing (Table 5).

**Table 5:** Performance of the XGBoost model with different variables

Variables	Number of variables	Accuracy [95% Cl]	Precision [95% Cl]	Recall [95% Cl]	F1 [95% Cl]	AUC [95% Cl]
Urine	15	0.862 ± 0.005 [0.855, 0.869]	0.839 ± 0.010 [0.825, 0.853]	0.862 ± 0.005 [0.855, 0.869]	0.832 ± 0.008 [0.822, 0.841]	0.788 ± 0.006 [0.780, 0.796]
Bilirubin	16	0.865 ± 0.004 [0.860, 0.871]	0.845 ± 0.007 [0.836, 0.855]	0.865 ± 0.004 [0.860, 0.871]	0.839 ± 0.005 [0.832, 0.846]	0.802 ± 0.006 [0.793, 0.810]
Albumin	17	0.864 ± 0.006 [0.856, 0.872]	0.843 ± 0.010 [0.829, 0.857]	0.864 ± 0.006 [0.856, 0.872]	0.838 ± 0.008 [0.827, 0.850]	0.805 ± 0.006 [0.797, 0.814]
BUN	18	0.866 ± 0.004 [0.861, 0.872]	0.847 ± 0.007 [0.837, 0.856]	0.866 ± 0.004 [0.861, 0.872]	0.842 ± 0.006 [0.834, 0.850]	0.811 ± 0.005 [0.804, 0.818]
Glu	19	0.867 ± 0.004 [0.860, 0.872]	0.846 ± 0.007 [0.836, 0.856]	0.867 ± 0.004 [0.860, 0.872]	0.841 ± 0.006 [0.832, 0.850]	0.811 ± 0.004 [0.805, 0.816]

### External validation

In our paper, we used the same indicator but a completely independent data set to externally verify the prediction performance of XGBoost. The data included 7 328 ICUs patients between 2014 and 2015, selected from the eRI database. The specific data distribution of the validation database was shown in Table 2.

### Evaluation

As the K-fold cross validation was used to avoid overfitting, we took the most popular one of K = 5. The data was divided equally to 5 folds, 4 folds were used for training and the remaining one fold was for evaluation. As the data distribution is unbalanced, accuracy is not a reliable measurement of prediction model performance [37]. In this paper, we used weighted averaged F1, recall, and precision to measuring model performance, which are defined as follows:

3 $${\text{Accuracy}} = \frac{{{\text{TP}} + {\text{TN}}}}{{{\text{TP}} + {\text{TN}} + {\text{FP}} + {\text{FN}}}}$$

4 $${\text{Recall}} = \frac{{{\text{TP}}}}{{{\text{TP}} + {\text{FN}}}}$$

5 $${\text{Precision}} = \frac{{{\text{TP}}}}{{{\text{TP}} + {\text{FP}}}}$$

6 $${\text{F}}1 = \frac{{2{\text{*Precison*Recall}}}}{{{\text{Precision}} + {\text{Recall}}}}$$

Note also that the prediction accuracy cannot be the only yardstick to select a classifier. Other criteria such as the Receiving Operating Characteristic (ROC) curve and the AUC are also used as metrics to measure the performance of prediction models.

## Results

### Optimization of prediction models

Table 3 shown the adjusted parameters and final values. For the hyperparameters of each classification algorithm, we first perform Grid Search with fivefold cross validation within the training set to determine the optimal hyperparameters. We then used the entire training set to train the model with the optimal hyperparameters and evaluated the trained model in the test set. For SVM classification, we tuned the parameters based on gamma and “C”; for LR classification, we tuned the parameters based on “C”; for NB, we tuned the parameters based on alpha; for ANN, we tuned the parameters based on hidden_layer_sizes; for RF classification, we tuned the parameters based on n_estimators; for XGBoost, we tuned the parameters based on learning_rate, n_estimators, gamma, reg_lambda, subsample, min_child_weight and max_depth.

**Fig. 5:**
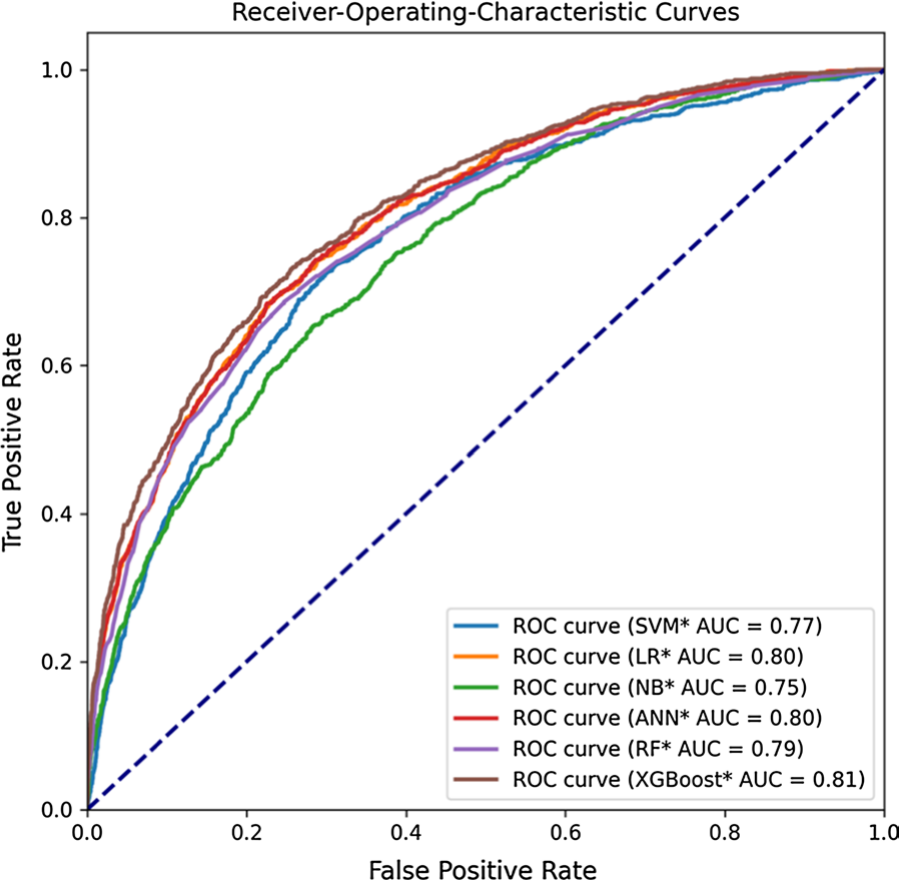
Receiver-Operating Characteristics curves of 6 models. * The data set used for model building contains 19 feature variables

As can be seen from Table 4, when the features were completely consistent with the Apache II scale (using 14 attribute features as input variables), none of the classification models had an AUC greater than 0.80. According to Fig. 4, we added five features successively to expand the features of the data set. The results presented in Table 6 and Fig. 5 shown that the performance of the XGBoost classifier has been gradually improved.

**TABLE 6:** Performance of optimized ML methods

Abbreviation	Accuracy [95% Cl]	Precision [95% Cl]	Recall [95% Cl]	F1 [95% Cl]
SVM*	0.774 ± 0.003 [0.770, 0.779]	0.826 ± 0.004 [0.820, 0.832]	0.774 ± 0.003 [0.770, 0.779]	0.794 ± 0.003 [0.790, 0.798]
LR*	0.739 ± 0.004 [0.734, 0.744]	0.840 ± 0.004 [0.834, 0.846]	0.739 ± 0.004 [0.734, 0.744]	0.770 ± 0.003 [0.766, 0.775]
NB*	0.840 ± 0.005 [0.833, 0.847]	0.814 ± 0.007 [0.805, 0.824]	0.840 ± 0.005 [0.833, 0.847]	0.822 ± 0.006 [0.815, 0.830]
ANN*	0.863 ± 0.004 [0.856, 0.869]	0.841 ± 0.009 [0.829, 0.853]	0.863 ± 0.005 [0.856, 0.869]	0.832 ± 0.007 [0.823, 0.841]
RF*	0.857 ± 0.003 [0.853, 0.862]	0.859 ± 0.003 [0.854, 0.863]	0.859 ± 0.003 [0.855, 0.864]	0.860 ± 0.004 [0.855, 0.865]
XGBoost*	0.867 ± 0.004 [0.860, 0.872]	0.846 ± 0.007 [0.836, 0.856]	0.867 ± 0.004 [0.860, 0.872]	0.841 ± 0.006 [0.832, 0.850]

^*^ The data set used for model building contains 19 feature variables

### Performance of 6 prediction models

The goal of the present study was to assess whether an ensemble ML technique, which would offer any gain in predicting hospital mortality in critically ill patients. In this paper, the mean and standard deviation of Accuracy, Precision, Recall, F1 and AUC values were used to evaluate the test results of each model. As you can see from Table 4, the accuracy of the APACHE II system in predicting actual mortality was limited. Combined with Table 4 and Fig. 3, the XGBoost model performed the best among all the models, with all evaluation indexes higher than the other models (0.858, 0.833, 0.859, 0.826 and 0.76, respectively).

For the sake of providing a fair comparison of our models with the Apache II, we included the same explanatory variables as used in Apache II. Expanding the set of explanatory variables used could potentially result in a score with even better predictive performance. For this reason, we added five new variables to build a new dataset as the new model dataset. The accuracy, precision, recall, F1 and AUC values of all models results of the new data set were shown in Table 6 and Fig. 5, the XGBoost model still has the greatest AUC value among all models, followed by the SVM, ANN and LR models. XGBoost and ANN models also have the greatest recall and precision values among all models in the MIMIC III data set. This was especially true for XGBoost.

### Feature importance analysis

**Fig. 6:**
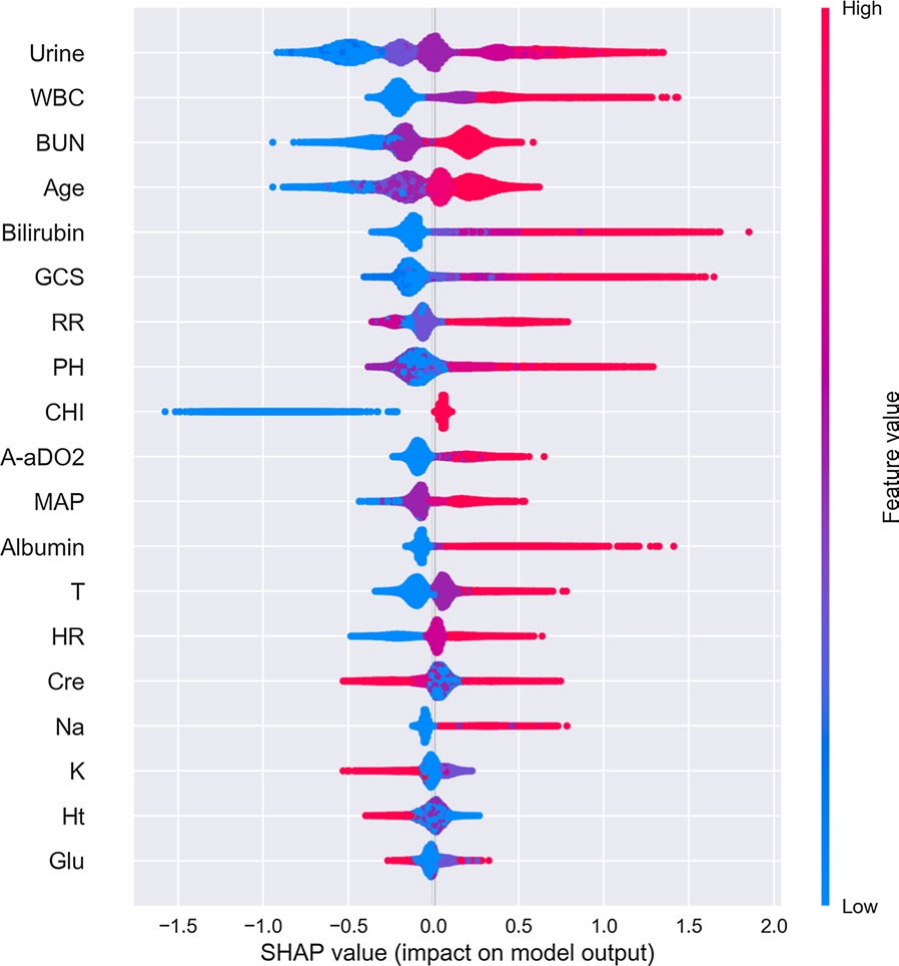
Feature importance of best factors combined from patients. Every patient has one dot on each row. The x position of the dot is the impact of that feature on the model’s prediction for the patient, and the color of the dot represents the value of that feature for the sample. Dots that don’t fit on the row pile up to show density. Since the XGBoost model has a logistic loss the x-axis has units of log-odds (Tree SHAP explains the change in the margin output of the model)

The Fig. 4 sorted by mean (|Tree SHAP|) below shown the urine feature as the strongest predictor of our XGBoost prediction model. And the top five feature vectors were urine, WBC, BUN, age and bilirubin, among which two variables were not included in the Apache II system. In addition, by plotting the impact of feature on each sample in Fig. 6, we can also get important outlier effects. For example, while bilirubin was not the most important feature globally, it was by far the most important feature for a subset of patients. The coloring by feature value shown us patterns such as patients with fewer chronic diseases have a lower risk of death, while higher WBC levels increase the risk.

### Results of external validation

**Fig. 7:**
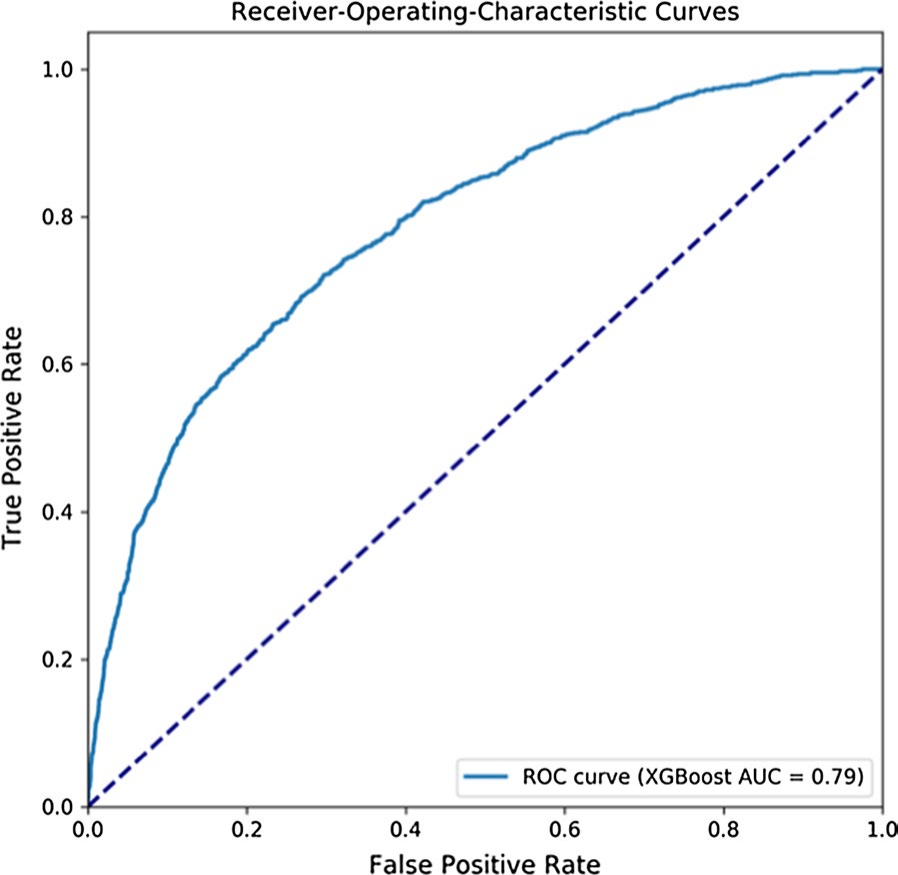
Receiver-Operating Characteristics curves of XGBoost, the data set comes from eICU

Our study also selected a set of patients hospitalized from the eICU database used for validation of our models. The ROC curve for XGBoost model-based prediction was provided in Fig. 7. The corresponding AUC was 0.79. The values of accuracy, precision, recall, and F1 were 0.812, 0.789, 0.809, and 0.791 respectively, indicating our prediction model can perform well in different data sets.

## Discussion

The significance of Apache II is the ability to quickly and accurately assess the current status of ICUs patients [38]. This has important implications for doctors' decision making after the patients are admitted. However, from Table 4 and Fig. 3, we found that Apache II did not perform well, worse than most machine learning models. The out-performance by ML methods shown promise in improving the quality of predictions. Similar to parts of the aims of this study, many researches used MIMIC III database to establish machine learning models for early disease prediction, but most of them were based on the prediction of single diseases with relatively small data volume [39–41]. For example, Layeghian et al. [19] used 4,611 samples in the MIMIC III database to establish a machine learning model for predicting cardiac arrest in patients with sepsis, the final accuracy and AUC were 0.76 and 0.82, respectively. Zhang et al. [40], through two machine learning algorithms, focused on 21 elements, including age, creatinine, BUN, and albumin, and was able to get an AUC of 0.86 when predicting volume responsiveness in patients with oliguric acute kidney injury. In our research, we used 24 777 cases for modeling and 7 328 samples for external verification, and the results shown that the accuracy, precision, recall, F1 and AUC of the model were all higher than 0.80.

We also seek to optimize Apache II scoring system by tuning parameters and expanding the dataset with available variables in our work. As shown in Table 5, the AUC of the XGBoost model increase from 0.76 to 0.81 may seem marginal, however, the AUC was approached only asymptotically, such an apparently small increase, in fact, represents a very large increase in model performance at these levels [42]. We have deliberately constrained our available variables to Apache II at first. It was interesting that we achieved a very impressive classification accuracy. After tuning and optimizing of the models, we added five new variables and found that the predictive ability of these models, especially XGBoost, was remarkable, the accuracy and AUC were substantially higher than other models. Moreover, the external verification results shown that the XGBoost model has good stability, it suggested that it could be used to develop a new prognostic clinical tool, and it was likely to succeed beyond the current Apache II scoring system.

In the end, considering that the included variables contribute differently to the model, we used the SHAP method called TreeSHAP, which is specifically for tree models. TreeSHAP can calculate the SHAP values of the corresponding features for all samples, and take their average value as the importance value of the feature to obtain the global interpretation [43]. The summary plot of 19 features in the XGBoost model according to their mean SHAP values was provided in Fig. 4. The global feature importance gives an abstract view about the role of each feature, but we cannot know the direction of these effects. So we then used the SHAP values to visualize the nonlinear relationship between risk factors and mortality risk in ICUs patients, see Fig. 6. However, the calculated data used in this paper were stratified by the Apache II scoring system, that is, the greater the difference between the value of characteristic variables and the normal value, the higher the score would be. The plot provided an estimate of both individualized and global feature importance by plotting the SHAP values for a random sample of instances. We found that higher bilirubin and GCS had a more significant impact on our model compared to urine. And most of the indicators have a positive effect on the risk of death, this result was broadly consistent with the medical literature [44–47]. Also, we notice that within a certain range, patients with higher levels of potassium (K) and hematocrit (Ht) had a lower risk than those with lower levels. We did not have enough continuous data to prove this, but it would be an interesting area to examine in the future.

From what has been discussed above, the predictive models based on machine learning have advantages in handling high-dimensional data, which suggests that more clinical variables can be considered as model inputs than those used in Apache II severity scoring systems, with the benefit of discovering meaningful clinical variables that have prediction effects on ICU mortality. In addition, our model’s strengths include its ability to predict the risk of death for all patients admitted to the ICU, rather than for a single disease.

There were some limitations to this study which have been mitigated, but not entirely avoided. First, while the data is of good quality for a clinical data set, it has drawbacks. The proportion of missing data was high for some variables, such as A-aDO2, bilirubin, and albumin. It was mitigated by using RF method to impute these missing data to preserve the sample size for the analysis. Second, clinical lab data, such as liver function, were omitted because in this study we chose features that were widely used in disease assessment scales and were known to predict patient mortality, which could yield good results according to this study. Future studies can be performed to determine whether data sets with more other features will produce comparable results. Third, some variables needed to compute the Apache II (e.g., elective surgery, underlying disease variables) were not directly available in the dataset and had to be extrapolated from other data. Fourth, our model was based on a retrospective study of two databases, which reduces its external validity. Hence, prospective validation is required for enabling the clinical implementation of our results.

## Conclusion

ICUs prognostication is important for clinical decision making, and the Apache II scoring system remains the most commonly used international severity scoring system worldwide [48]. We have demonstrated that Apache II is not the most suitable approach and it can be augmented by changing the calculation method and adding the physiological parameters. We also presented a prediction model based on XGBoost algorithm, along with proofs and experiments showing that model was desirable. Promising next steps is to further improve the prediction performance of model by expanding the number of variables, and carry out prospective validation work.

## Data Availability

The datasets supporting the conclusions of this article are available in the https://mimic.physionet.org/ and https://eicu-crd.mit.edu/.

## Abbreviations

A-aDO_2_: Alveolar-arterial differences for O_2_
AIDS: Acquired immune deficiency syndrome
ANN: Artificial neural networks
APACHE: Acute physiology and chronic health evaluation
APS: Acute Physiology Score
AUC: Area under the curve
BIDMC: Beth Israel Deaconess Medical Center
BUN: Blood urea nitrogen
CHI: Chronic Health Index
Cre: Serum creatinine
COPD: Chronic obstructive pulmonary disease
eRI: EICU Research Institute Database
GCS: Glasgow Coma Scale
HR: Heart rate
Ht: Hematocrit
ICUs: Intensive care units
K: Potassium
LODS: Logistic Organ Dysfunction Score
LR: Logistic regression
MAP: Mean arterial pressure
MIMIC-III: Medical Information Mart for Intensive Care version III
ML: Machine learning
MODS: Multiple Organ Dysfunction Score
Na: Sodium
NB: Naïve Bayes
PH: Potential of hydrogen
RF: Random forest
ROC: Receiving operating characteristic curve
RR: Respiratory rate
SAPS: Simplified Acute Physiology Score
SOFA: Sepsis-related Organ Failure Assessment Score
SVM: Support vector machine
T: Temperature
WBC: White blood cell
XGBoost: EXtreme gradient boosting
